# Clinical Impact of the Use of Ologen in Filtering Surgery Performed in Uncontrolled Glaucoma

**DOI:** 10.3390/jcm13154463

**Published:** 2024-07-30

**Authors:** José-Manuel Navero-Rodríguez, Júlia Boldú-Roig, Laura Pinilla, María Vidal-Martí, Alfonso Antón

**Affiliations:** 1 Institut Català de Retina, Glaucoma Department, 08022 Barcelona, Spain; 2 Department of Ophthalmology, Universitat Internacional de Catalunya, 08017 Barcelona, Spain; 3 Department of Ophthalmology, Hospital Universitari Bellvitge, 08907 Barcelona, Spain

**Keywords:** Ologen, collagen matrix implant, filtering surgery

## Abstract

**Introduction**: To compare the efficacy and safety of trabeculectomy with a collagen matrix implant (Ologen^®^) versus trabeculectomy with mitomycin C (MMC) versus trabeculectomy with both Ologen^®^ and MMC (OLO + MMC). **Methods:** This non-randomized study included 119 eyes of 101 patients with uncontrolled open-angle glaucoma who underwent trabeculectomy, either alone or combined with phacoemulsification. The data were initially recorded following a standard surgical protocol, using an electronic database with structured fields. The patients were divided into three groups: 44 received trabeculectomy with adjunctive MMC (MMC group), 34 received surgery with Ologen^®^ (OLO group), and 41 received surgery with both Ologen^®^ and MMC (OLO + MMC group). The main outcome measures were the change in intraocular pressure (IOP), change in number of medications needed, complete success rate (defined as IOP ≤ 20 mmHg and at least 20% IOP reduction without hypotensive medications), rate of complications, and rate of postoperative interventions. The follow-up period was 36 months. **Results**: IOPs significantly decreased (*p* = 0.01) in all groups across all study visits, decreasing from 19.8 ± 4.6 mmHg to 12.7 ± 4.2 mmHg in the MMC group, from 20.5 ± 4.7 mmHg to 13.9 ± 3.5 mmHg in the OLO group, and from 23.5 ± 6.1 mmHg to 13.1 ± 3.5 mmHg in the OLO + MMC group. After correcting for the baseline IOP, only the first two postoperative visits (first week and first month) showed a significantly greater IOP reduction in the OLO + MMC group. The number of hypotensive medications was significantly reduced from 3.1 ± 0.6 to 0.56 ± 1.1 in the MMC group, from 2.9 ± 0.4 to 0.83 ± 1.1 in the OLO group, and from 3.0 ± 0.6 to 0.45 ± 0.95 in OLO + MMC group, with no statistically significant differences among the groups (*p* = 0.57). The complete success rates were 63.6% in the MMC group, 67.6% in the OLO group, and 80.5% in the OLO +MMC group, with no statistically significant differences between the groups (*p* = 0.21). Suture release was significantly more frequent in the MMC group (86.1%) than in the OLO group (62.1%) and in the OLO + MMC group (45.9%; *p* = 0.02). Bleb needling, with (33.3%; *p* = 0.005) or without (66.7%; *p* = 0.0001) 5-fluorouracil injection (5-FU), was significantly more common in the MMC group. The highest complete success rate (61%) was observed in the OLO + MMC group. **Conclusions**: The use of Ologen^®^ and mitomycin C provided similar surgical IOP reduction in glaucoma surgery compared with either MMC or Ologen^®^ alone, but significantly reduced the need for postoperative interventions.

## 1. Introduction

Trabeculectomy has been the gold-standard glaucoma surgery since it was described by Cairns in 1968 [1]. Episcleral and subconjunctival scarring is the main cause of failure of filtering surgery. The intraoperative use of antimetabolites, mitomycin C (MMC), and 5-fluorouracil (5-FU), as well as postoperative handling of sutures, needling, or 5-FU injections, have allowed surgeons to modulate and decrease conjunctival scarring as well as to improve filtering surgery success [2,3]. Indications for needling are encapsulation, scarring blebs with slow filtration, or insufficiently low intraocular pressure (IOP) [4]. Currently, surgeons are still searching for substances and/or implants and/or postoperative maneuvers that could modulate conjunctival scarring, to increase the efficacy and survival rate of filtering surgery and, more importantly, to customize IOP results to suit each patient’s needs.

Ologen^®^ (Aeon Astron Europe BV, Leiden, The Netherlands; OLO) is a matrix of biodegradable collagen (CM), composed of a crosslinking of type I atelocollagen at more than 90% and glycosaminoglycans at less than 10%. It is a three-dimensional structure with porous diameters ranging from 10 to 300 microns. It has been proposed that this matrix acts on three different levels that may optimize the effect of MMC (inhibiting fibroblast proliferation). Firstly, it induces a random growth of fibroblasts and collagen fibers. Secondly, it behaves as a spacer between the conjunctiva–tenon and the episclera. Finally, it becomes a reservoir for aqueous humor. We hypothesize that the addition of MMC and a collagen matrix may not only have effects on IOP, but also on postoperative management and complications. The three mechanisms of action of the collagen matrix could potentiate the anti-scarring effect of MMC as well as facilitate a slower flow that could improve the bleb formation process.

Previous studies comparing the efficacy of trabeculectomy with an Ologen^®^ implant vs. adjunctive mitomycin C at different concentrations have reported conflicting results. Generally, there are no statistically significant differences after 12 months in terms of efficacy, medication reduction, and complications [5,6,7].

In 2018, Castejon et al. studied the combination of Ologen^®^ and MMC at low doses (0.1 mg/mL) in patients undergoing trabeculectomy and phacotrabeculectomy with 2 years of follow-up. They obtained equal results in the trabeculectomy group both with and without Ologen^®^, but better IOP results when using Ologen^®^ in the phacotrabeculectomy group [8].

There is no current study comparing Ologen combined with MMC at the dose of 0.2 mg/mL vs. either Ologen or MMC alone. The present study intends to compare the efficacy and safety of adjunctive MMC, Ologen^®^, and both together, and the required postoperative maneuvers in glaucoma patients requiring filtering surgery.

## 2. Patients and Methods

### 2.1. Study Design

This is a comparative and retrospective study with systematic data collection following a predefined surgical protocol. This study was approved by the Institutional Review Board of our institution and adhered to the principles outlined in the Declaration of Helsinki.

The medical histories of patients operated on consecutively by the same glaucoma expert surgeon (JN) after 2011 were retrospectively reviewed. One hundred and nineteen eyes of 101 subjects were consecutively included after verifying the inclusion criteria. All of them underwent filtration surgery, with or without simultaneous phacoemulsification. All patients were Caucasian.

The patients were divided into 3 groups. The MMC group comprised 44 eyes that underwent filtering surgery (trabeculectomy) before 2014 with intraoperative MMC (0.2 mg/mL for 2 min). The OLO group included 34 eyes that underwent filtering surgery (trabeculectomy) between 2014 and 2016, with a subconjunctival Ologen^®^ CM implant, model 830,601 (12 mm × 1 mm), at the end of surgery. Finally, the OLO + MMC group comprised 41 eyes that underwent trabeculectomy between 2016 and 2019 using the same MMC dose and time as group I, and subconjunctival Ologen^®^. We included patients treated consecutively, before and after the Ologen^®^ implant was introduced in our clinical practice. In summary, all those patients having surgery before January 2014 did not have the implant. After that, every patient received the Ologen^®^ implant. According to the diagnosis, the patients were divided into 3 groups, namely primary open-angle glaucoma (POAG), pseudoexfoliative glaucoma (PEXG), and chronic angle closure glaucoma (CACG). The POAG group included 96 eyes, the PEXG group included 15 eyes, and the CACG group included 8 eyes. Additionally, the three diagnostic groups were analyzed separately with ANOVA.

The inclusion criteria were the following: diagnosis of primary open-angle glaucoma, pseudoexfoliative glaucoma or chronic angle closure glaucoma and inadequate IOP control in the opinion of the ophthalmologist who followed the patient and performed the filtering surgery (JN).

Of the 119 eyes, 64 underwent phacotrabeculectomy, all of which had the additional diagnosis of cataract with visual acuity under 20/25. Exclusion criteria were the following: diagnosis of any other type of secondary glaucoma (post-traumatic, neovascular, aphakic…), previous vitro-retinal surgery, or a follow-up period of less than 3 years. Additionally, all cases with posterior capsule rupture, vitreous prolapse in the anterior chamber, or impossible IOL implantation in the bag were excluded.

### 2.2. Examinations

Patients were evaluated preoperatively and postoperatively at 1, 7, 14, 21, and 30 days, and 3, 6, 12, 18, 24, 30, and 36 months after the intervention, following a predefined surgical follow-up protocol. Each examination included measurement of Snellen best corrected visual acuity (BCVA), IOP measured with applanation tonometry, slit-lamp bio-microscopy, and fundus examination. Complications and postoperative interventions were recorded. Visual field (VF) testing was performed at a minimum of at least baseline, 12, 24, and 36 months, with the program SITA Standard 24-2 (Humphrey Visual Field, HFA II-i 740-15908/5.0, software version 3.1.1.264, Carl Zeiss Meditec, Dublin, CA, USA).

### 2.3. Definitions

Glaucoma was defined as the presence of simultaneous functional and structural damage. A glaucomatous VF defect was defined by the presence of a confirmed (in at least 2 VFs) group of three or more contiguous points with *p* < 5% and one of them with *p* < 1% in the pattern deviation map. The optic nerve was evaluated in color photographs and considered glaucomatous when there was rim thinning, rim notch, a papillary splinter hemorrhage, a nerve fiber layer defect, and a cup-to-disc ratio asymmetry more than 0.3 between two eyes that could not be explained by asymmetry in the optic disc size. Glaucoma severity was classified according to mean deviation (MD) using the Hodapp criteria [9]. Complete success was defined as IOP under 20 mmHg and at least 20% IOP reduction without hypotensive medications, and qualified success was considered to be when IOP was under 20 mmHg and IOP was reduced by over 20% using ocular hypotensive medications. Therapeutic failure was defined as an IOP over 20 mmHg or a reduction in IOP of less than 20% with medications, or an IOP under 6 mmHg on two consecutive study visits, or by the need for a second glaucoma surgery. The definitions for success and failure were defined following the Guidelines on Design and Reporting of Glaucoma Surgical Trials [10]. If IOP rose to a level considered too high by the physician (JN), anti-glaucoma medication was initiated. If medical treatment after surgery failed to lower IOP to an acceptable level, or if VF or structural damage progressed, glaucoma surgery was repeated.

### 2.4. Outcome Measures

The primary outcome was the mean change in IOP from baseline to the last follow-up visit. The secondary outcomes were the number and type of postoperative interventions (massage, suture release, needling, 5-FU injections), success rate, and frequency of complications.

### 2.5. Surgical Technique

An experienced cataract and glaucoma surgeon (JN) performed all surgeries using the same standardized technique. The surgical procedure consisted of peribulbar anesthesia and corneal traction sutures. Phacoemulsification was performed via a 2.2 mm clear-cornea incision in either the nasal or temporal quadrant with intraocular lens (IOL) implantation. Complete aspiration of the viscoelastic substance was performed at the end of phacoemulsification. The filtering procedure was placed to the right of the phaco-incision in the upper opposite quadrant. In all cases, dissection of a superior fornix-based conjunctival flap was performed. Electrocautery was used to control episcleral bleeding. A square (3 × 3 mm) scleral flap of half the scleral thickness was prepared. In the first two groups (MMC and OLO + MMC groups), MMC 0.2 mg/mL was applied using three sponges (4 × 4 mm) over the scleral flap and under the conjunctiva for 2 min, and washed with 50 mL of saline solution afterward. Excision of trabecular meshwork was performed with a Kelly punch. A peripheral iridectomy was excised in all cases. In all three groups, two releasable 10/0 nylon sutures were placed on both corners of the scleral flap. Then, the flow was checked; the anterior chamber was filled with saline solution. In the last two groups (OLO and OLO + MMC groups), a flat cylindrical OLO implant 12 mm in diameter and 1 mm in height (Ologen model no. 862051; Aeon Astron Europe BV, Leiden, the Netherlands) was placed over the scleral flap without suturing. Finally, a hermetic conjunctival suture was performed at the limbus with 10/0 nylon.

### 2.6. Postoperative Care

The standard postoperative regimen consisted of topical moxifloxacin three times a day for 7 days and dexamethasone every 2 h for the first month and gradually tapered off during the second and third months. At each visit, the investigators examined the eye and evaluated the need for bleb manipulations. Two types of bleb manipulation were considered. Mechanical maneuvers could be performed to increase flow and break down adhesions or scarring in the bleb. This included massage, removal of releasable sutures, and/or needling. Additionally, pharmacological bleb management could also be performed by using subconjunctival 5-fluorouracil injections (0.1 mL at 50 mg/mL concentration) around the bleb, to aid draining and prevent scarring. These procedures were recorded and were not considered treatment failures but postoperative interventions.

### 2.7. Statistical Analysis

The three treatment groups and the three diagnostic groups were compared with One-Way ANOVA (quantitative variables) and the Chi^2^ Test (qualitative variables).

The statistical analysis was performed with MedCalc Statistical Software version 20.013 (MedCalc Software Ltd., Ostend, Belgium; https://www.medcalc.org; accessed on 11 March 2021) and SPSS (IBM SPSS Statistics for Windows, IBM Corp. Released 2019, version 26.0; Armonk, NY, USA: IBM Corp).

## 3. Results

All patients underwent filtration surgery, with or without simultaneous phacoemulsification, at our center after 2011. Among the 119 eyes included in the study, 64 eyes (53.8%) underwent phacotrabeculectomy and 55 underwent trabeculectomy alone.

### 3.1. Baseline Patient Characteristics

The patient demographics are shown in Table 1. No statistically significant differences were found among the groups regarding age, sex, preoperative medication, type of glaucoma, or cup/disk ratio. The mean preoperative IOP was 19.8 ± 4.6 mmHg in the MMC group, and 20.5 ± 4.7 mmHg in the OLO group. These were significantly lower (*p* = 0.004) than the 23.5 ± 6.1 mmHg found in the OLO + MMC group. The MMC group had slightly worse BCVA (*p* = 0.04), Visual Field Index (VFI; *p* = 0.01), and median deviation (MD; *p* = 0.03) values than the OLO group and OLO + MMC group.

### 3.2. Postoperative Intraocular Pressure

**Figure A1:**
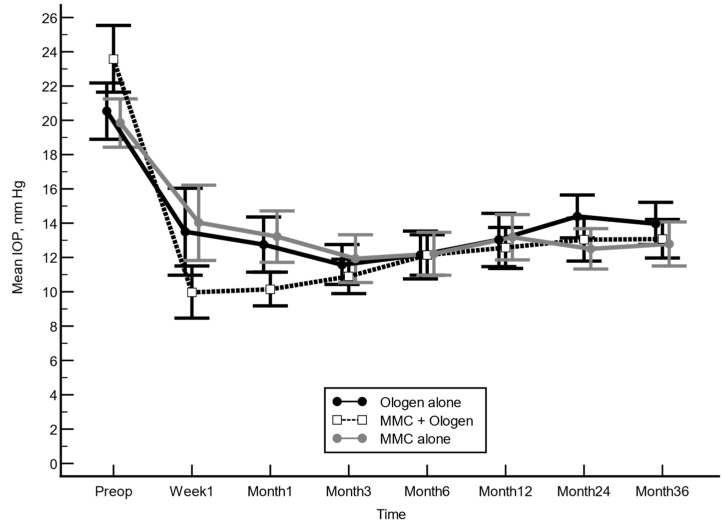
Mean IOPs by treatment group. IOP reduction from baseline was significant compared with baseline in all groups and at all time points. No significant differences were found in mean IOPs at any follow-up visits among the groups.

**Figure A2:**
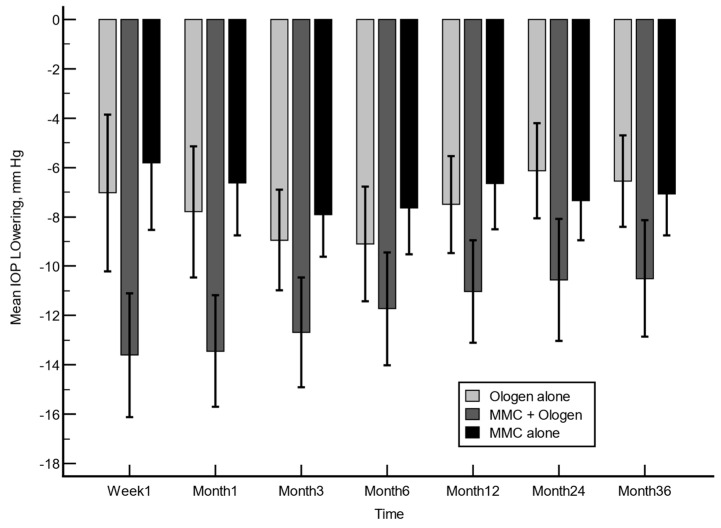
IOP reductions in all groups at different time points. Although IOP reduction was significantly greater in the OLO + MMC group, after adjusting for age, preoperative IOP, number of preoperative ocular hypotensive medications, type of surgery, and cataract surgery or not, as covariates, mean IOP reduction (absolute values) was significantly greater in the OLO + MMC group only at one week and one month (*p* > 0.05).

**Figure A3:**
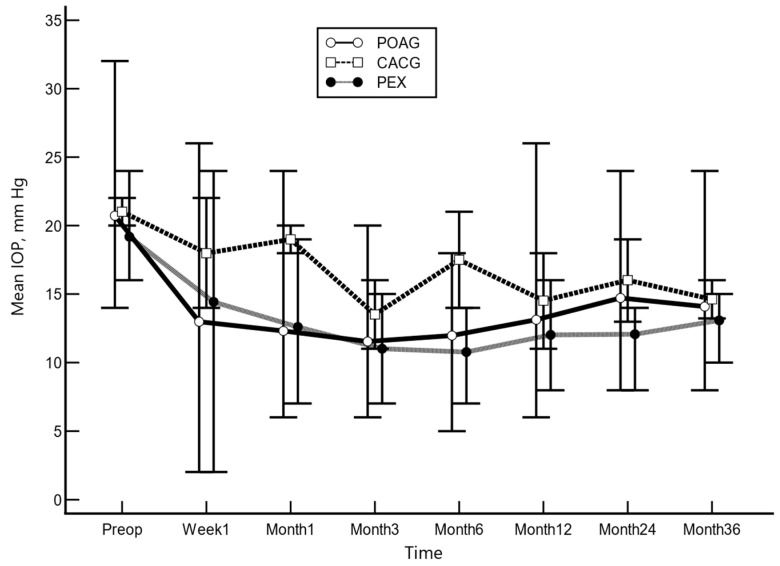
Mean IOP by diagnosis in the Ologen-alone group. No significant differences among the groups.

**Figure A4:**
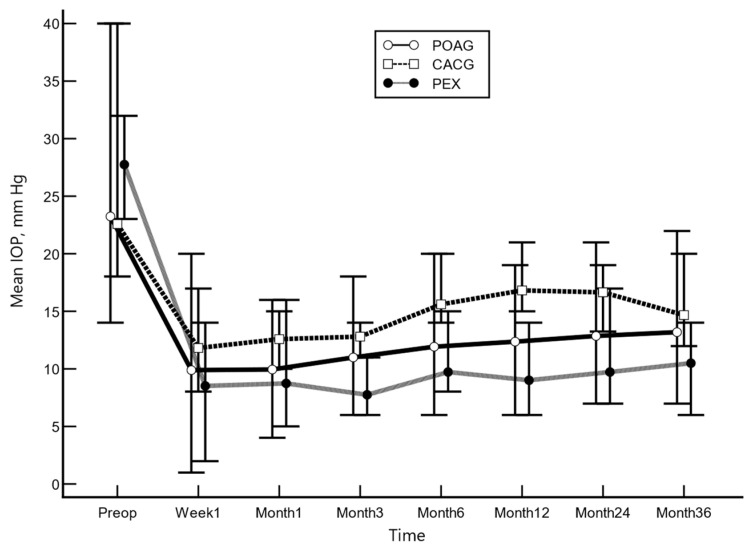
Mean IOPs by diagnosis in the MMC + Ologen group. No significant differences among the groups.

**Figure A5:**
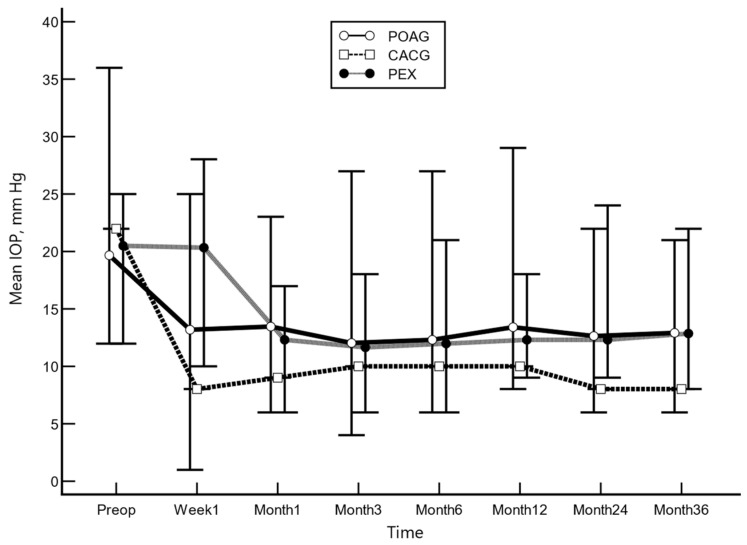
Mean IOPs by diagnosis in the MMC-alone group. No significant differences among the groups.

The IOP was significantly reduced after surgery in all three groups at all follow-up visits (*p* < 0.05; Figure A1). The average postoperative IOP (mean ± SD) was 12.7 ± 4.2 mmHg at 36 months, and it was under 15 mmHg in all groups at all time points. In the MMC group, the mean IOP was 13.2 ± 4.3 mmHg at 12 months, 12.5 ± 3.9 mmHg at 24 months, and 12.7 ± 4.2 mmHg at 36 months; in the OLO group, the mean IOP was 13.0 ± 4.4 mmHg at 12 months, 14.4 ± 3.6 mmHg at 24 months, and 13.9 ± 3.5 mmHg at 36 months; and, finally, in the OLO + MMC group, the mean IOP was 12.5 ± 3.7 mmHg at 12 months, 13 ± 3.9 mmHg at 24 months, and 13 ± 3.5 mmHg at 36 months. The IOP reduction was significantly greater in the OLO + MMC group at all visits, but after adjusting for age, preoperative IOP, number of preoperative ocular hypotensive medications, type of surgery, and simultaneous cataract surgery, as covariates, the mean IOP reduction (absolute values) was significantly greater in the OLO + MMC group only at one week and one month (*p* > 0.05), but not at any other time points. There were no statistically significant differences in IOP reduction between the trabeculectomy vs. phacotrabeculectomy eyes in the three groups at 36 months (*p* = 0.8). The IOP reduction in all groups is shown in Figure A2; the percentage of eyes with IOP below 14 mmHg was 72.7% in the MMC group, 61.8% in the OLO group, and 73.2% in the OLO + MMC group. The mean IOP reductions for the groups MMC, OLO and OLO+MMC by diagnosis of POAG, PEXG, and CACG are shown in Figure A3, Figure A4 and Figure A5.

### 3.3. Success Rate

**Figure A6:**
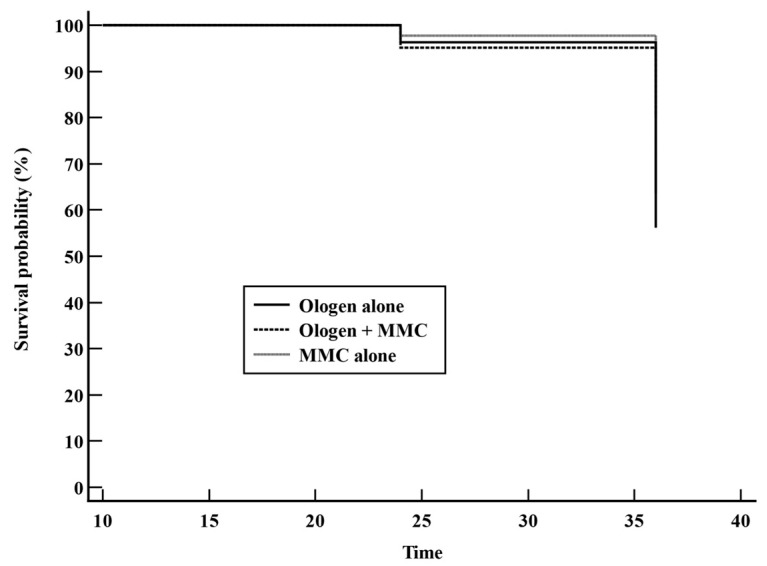
Kaplan–Meier survival curves (*p* < 0.13) for qualified success (IOP≤ 20 mmHg with at least one medication). Log-rank (Mantel–Cox test) *p* = 0.01.

At month 36, the overall success rate was 63.6% in the MMC group, 67.6% in the OLO group, and 80.5% in the OLO + MMC group. The complete success rates were 50% in the MMC group, 52.9% in the OLO group, and 61% in the OLO + MMC group. Finally, the qualified success rates were 13.6% in the MMC group, 14.7% in the OLO group, and 19.5% in the OLO + MMC group. No differences in Kaplan–Meier survival curves (*p* < 0.13) were found among the groups (Figure A6).

### 3.4. Number of Medications

There was a significant decrease in the number of medications required after surgery compared with baseline (*p* < 0.00) in each group. Considering the whole sample, there were no significant differences among the groups at any time point during the study. At baseline, the mean ± SD number of medications was 3.1 ± 0.6 in the MMC Group, 2.9 ± 0.4 in the OLO Group, and 3.0 ± 0.6 in the OLO + MMC group. At 36 months after surgery, the MMC group required an average of 0.5 ± 1 medications, the OLO group needed a mean of 0.7 ± 1.1 medications, and the OLO + MMC group required 0.4 ± 1 (*p* = 0.29) medications. Of the patients, 70%, 67%, and 76% were without medications at the end of the study in the MMC group, the OLO group, and the OLO + MMC group, respectively.

### 3.5. Postoperative Interventions

The three groups received postoperative interventions as needed during the follow-up period (Table 2). The total numbers of interventions were 74, 31, and 29 in the MMC group, the OLO group, and the OLO + MMC group, respectively. The frequency of bleb massage was not statistically different among the groups (*p* = 0.8). Suture release was performed significantly less frequently in the OLO and OLO + MMC groups (both with Ologen^®^) than in the MMC group (*p* = 0.02). Similarly, mechanical bleb needling (*p* = 0.016) and 5-FU injections (*p* = 0.000) were significantly more frequent in the MMC group than in the other two groups. Glaucoma re-operation was required in four patients in the MMC group and two in each of the OLO and OLO + MMC groups during the follow-up period.

### 3.6. Complications

Complications were defined as any deviation from the normal postoperative course. All complications are shown in Table 3. There were no intraoperative complications in any of the eyes in either group. A total of 90 eyes (75.6%) did not present any complications. There was no significant difference in the frequency of complications among the groups (*p* = 0.09). Transient hypotony was found in 11.4% (*n* = 5) of eyes in the MMC group, 17.6% (*n* = 6) in the OLO group, and 16.7% (*n* = 7) in the OLO + MMC group (*p* = 0.69). Only one eye in the OLO + MMC group developed hypotony with macular folds, which was managed by surgical intervention via placement of conjunctival compression sutures. No adverse reaction to the Ologen^®^ implant, matrix extrusion, or conjunctival erosion was observed.

### 3.7. Visual Acuity

As expected, patients who underwent phacotrabeculectomy manifested a significant improvement in visual acuity. There were no statistically significant differences in BCVA among the groups (*p* = 0.12).

## 4. Discussion

It is well known that episcleral fibrosis and subconjunctival scarring decrease the success rate of glaucoma surgery. For that reason, adjunctive antimetabolites have been used for decades to improve surgical efficacy [2,11]. Nevertheless, the complications of the use of MMC, such as hypotony, avascular blebs, or infection have led clinicians and researchers to look for new substances or materials that could offer similar efficacy with fewer complications.

Ologen^®^ was initially proposed as a possible substitute for MMC [5,7]. A recent systematic review and meta-analyses indicated that trabeculectomy with Ologen was a safe and effective procedure in patients with glaucoma, but it was also associated with less IOP lowering and fewer cases of hypotony than when trabeculectomy is augmented with MMC [5,12]. The present study combined the antifibrotic action of MMC with the capabilities of Ologen CM, which include separating the conjunctiva from the episclera, acting as a reservoir of aqueous humor, and, in addition, preventing the organization of fibroblast fibers. To the best of our knowledge, this is the first study that compares the outcomes of trabeculectomy and phacotrabeculectomy with Ologen (12 mm × 1 mm) together with MMC (0.2 mg/mL) versus trabeculectomy with Ologen only, versus trabeculectomy with MMC only.

Evaluating surgical techniques is always challenging due to the constantly evolving surgical options, the large volume of publications (many of which carry a considerable risk of bias), and the necessity of long-term follow-up, since short-term results hold little significance in chronic, lifelong diseases. Moreover, any treatment should aim to improve the patient’s clinical status and/or prognosis while minimizing avoidable harm.

The present study associated and compared two well-known varieties of trabeculectomy for three years and obtained significant results that could be applied in clinics. Nevertheless, this study has certain limitations, which we believe do not impede achieving its objective. First, it is a retrospective study, but patients were examined following a predefined surgical and postoperative protocol, and parameters were recorded on a structured electronic database. Second, only one center and one surgeon participated, limiting the strength but enhancing the homogeneity of the procedures and examinations. Third, the distribution of patients in the treatment group was not randomized. Rather, patients were operated on with one of the three techniques selected consecutively, with all the MMC patients recruited first, then all those in the OLO group, and over the recent years, cases were allocated to the OLO + MMC group. Finally, a limited and relatively low number of cases were included. However, there was long-term follow-up, which allowed the identification of significant differences among the groups.

One difficulty in comparing surgical results is the differences in the techniques used in the studies. With Ologen CM, there is an added potential influencing factor, which is the size and height of the matrix. In our study, a circular 12 mm × 1 mm matrix was used. Narayanswam used a circular 7 mm × 4 mm matrix, and Cillino and many other authors used a 6 mm × 2 mm matrix [7,13]. The lower total volume of the Ologen matrix could partially account for the lower final IOP values obtained in their studies [7]. We speculate that the Ologen 6 mm (D) × 2 mm (H) matrix (model: 830601) covers too small an area, raises the height of the filtration bleb due to its 2 mm height, and could also exert pressure on the scleral flap, hindering the outflow of aqueous humor. While the latter could explain why some authors find a lower frequency of hypotony in the group treated with Ologen [14], we preferred the use of the Ologen 12 mm (D) × 1 mm (H) matrix (model: 862051), which allowed us to cover a larger surface and facilitated the formation of a flatter, more diffuse and posterior bleb, while exerting less pressure on the scleral flap.

We found no significant differences in mean IOP among the groups at any time point. The IOP values were similar to those of Castejon et al., who used MMC 0.1 mg/mL, and slightly lower than those obtained by Cillino at 24 months. That study reported IOPs over 16 mmHg [8,15]. Differences could be related to technique (different Ologen size) and/or the characteristics of the sample, among other causes.

The present study identified a greater IOP reduction in the OLO + MMC group. After adjusting for age, preoperative IOP, number of preoperative ocular hypotensive medications, type of surgery, and the presence of simultaneous cataract surgery, as covariates, the mean IOP reduction (absolute values) was significantly greater in the group treated with Ologen + MMC only at one week and one month, but not at other study time points.

All groups showed a significant and similar decrease in the medications needed, with values similar to those reported by Sen et al. [16], who found a decrease in required medications from 3 to 0.5 with MMC or with OLO. Our results showed a non-significant tendency toward a higher value in the final postoperative medications needed in the OLO group (0.7) compared to the OLO + MMC group (0.4). Similar figures for changes in medication required were found by Elwehidy et al., who compared trabeculectomy augmented with Ologen vs. perfluoropropane gas, and observed a reduction from 3.2 to 0.5 in the Ologen group at 36 months [17].

Success rates can be very useful when evaluating and comparing different techniques. However, it is important to check the definitions used in each study to compare results from different manuscripts. Cillino obtained a rather low success rate of 40% at 24 months with MMC or with Ologen. This could be explained partially by the demanding definition of success being an IOP under 15 mmHg, but also by the slightly higher mean final IOPs reported [15]. Min et al. studied the use of Ologen soaked with MMC (0.1 mL of 0.2 mg/mL) prospectively in 30 eyes and found, at 12 months, a complete success rate of 40%, with an identical definition applied in the present study [14]. This value is lower than the 61% at 36 months obtained by the Ologen + MMC group in our study. Additionally, we found IOPs < 14 mmHg without medication in 72.7% of the MMC group, 61.8% in the Ologen group, and 73.2% in the Ologen + MMC group at 36 months. These figures are again superior to those reported by Cillino et al. at 24 months [15]. In their study, IOP was under 15 mmHg in 40% of the MMC group and 50% of the Ologen group [15].

Postoperative maneuvers, whether mechanical or medical, are very important and frequently required to modulate bleb scarring. For that reason, they become a significant factor that should be evaluated as an outcome measure of the different techniques. Suture manipulation can be very useful in the early postoperative period, to increase flow when needed. One important issue is that the Ologen matrix does worsen external visual access to sutures, and this needs to be taken into account in the design of the surgical technique. Additionally, the use of thicker Ologen implants increases the difficulty of seeing and reaching sutures with the laser and undoubtedly increases bleb height for at least several months. The use of releasable sutures from the corneal surface, thinner Ologen models, or modeling an area of the Ologen to leave part of the sutures visible can facilitate suture manipulation. Cillino et al. [16] obtained similar IOP values with MMC or Ologen when leaving one of the sutures untied in the Ologen group. Narayanswam et al. [13] used only one fixed suture with minimal tension and one releasable suture in the OLO group and two fixed sutures in the MMC group, whereas we sutured all flaps with two releasable sutures. All these slight differences in technique might influence the global results, particularly those related to the number of suture lyses or suture releases performed. The higher percentage of suture release maneuvers in our study was probably related to the fact that two sutures were used. This could also be the reason for the low final IOPs we found, with a global mean value of 12.9 mmHg. In our study, suture release was needed less frequently in the OLO (62%) or OLO + MMC (47%) groups than in the MMC group (86%). This suggests that Ologen helps flow maintenance and keeps IOP sufficiently low to require less suture release. This could be explained by the Ologen action of keeping space between the sclera and conjunctiva and acting as a reservoir for aqueous humor. The use of a releasable suture or 1 mm height Ologen is recommended to facilitate access to sutures with the laser.

Bleb needling was performed in 27% of the patients in the MMC group, while in the OLO and OLO + MMC groups, it was performed in only 7% and 13% of the cases (*p* = 0.016), suggesting that Ologen may reduce the risk for bleb encapsulation. Nevertheless, Cillino et al. Did not found significant differences with reported bleb needling of 35% in the MMC group and 30% in the OLO group. Naryanswam reports 40% bleb needling in the OLO group and 6% in the MMC group [7,13]. The latter difference could be explained by the fact that their study used a higher concentration of MMC (0.4 mg/mL to 2 min) and a low rate of suture lysis in the Ologen group. These contradictory results could be related to the different study criteria used to indicate bleb.

Subconjunctival 5-FU injections in the postoperative period were performed more frequently in the MMC group (66%) than in the OLO (14%) or OLO + MMC groups (16%, *p* < 0.001). Similarly, Papaconstantinou [18] reports an occurrence of 10% in the Ologen group and 25% in the MMC group, although this difference was not significant. Our results indicate that postoperative management was easier in those patients who were operated on using Ologen, with fewer procedures required. As many as 74 postoperative interventions were performed in the MMC group, while in the groups where Ologen was used, only 29 and 31 maneuvers were needed.

Additionally, the three diagnostic groups were also analyzed separately with ANOVA. The results from those patients with POAG alone confirmed all previous findings from the sample as a whole. The IOP values were very similar, and the use of MMC and Ologen together decreased the need for postoperative interventions. The comparisons among the three groups do not show any statistical differences among the groups in IOP, the need for hypotensive medications, or postoperative interventions. Nevertheless, although it seems that all three groups behaved similarly, the sizes of the PEXG and CACG groups were not big enough to solidly conclude that there are no clinically significant differences among them.

The degree and speed of degradation of the collagen matrix during the postoperative period remain unknown. From a clinical perspective, it seemed that the collagen integrated with the scarring tissue over the course of several months. Although the collagen matrix could be seen through the conjunctive of the bleb for a few weeks, it soon disappeared under the bleb’s wall, and it was not possible to determine if there was any of it remaining. Sequential OCT images could possibly help to describe this process. Unfortunately, OCT images of the bleb were not performed during the study.

The use of Ologen in filtration surgery seems to be at least as safe as traditional trabeculectomy. The only complication that appeared more frequently in our study in the OLO + MMC group was wound leak (*p* = 0.02). This has also been reported by other authors, such as Cillino et al. [15] and Tanna et al. [19]. It is possible that the association of MMC that delays the healing process with the expanding effect of Ologen may increase the tendency to present wound leak. These results recommend paying special attention to obtaining a water-tight conjunctival closure. Finally, in our study, no allergy or increased inflammatory reaction was reported for up to 36 months.

In summary, the use of an Ologen implant in conjunction with MMC in trabeculectomy does not seem to influence final IOP values, whilst it facilitates postoperative care by reducing the need for suture release, 5-FU injections, or bleb needling. Prospective, randomized, long-term studies are needed to confirm this result, as well as to evaluate if it might improve bleb survival over time.

## Figures and Tables

**Table 1:** Baseline patient characteristics in the MMC, OLO, and OLO + MMC groups.

	Units	MMC	OLO	OLO + MMC	*p*
Age in years	(mean ± SD)	67.8 (11.2)	71.5 (13.6)	68.9 (12.3)	0.41
Gender	(M/F)	22/22	14/20	18/24	0.69
BCVA	(mean ± SD)	0.69 (0.2)	0.79 (0.2)	0.8 (0.2)	0.04
Type of glaucoma					0.96
POAG *	*n* (%)	35 (79.5)	21 (79.4)	32 (76.2)	
CACG *	*n* (%)	3 (6.8)	2 (5.9)	5 (11.9)	
PEXG *	*n* (%)	6 (13.6)	5 (14.7)	5 (11.9)	
Preoperative IOP	(mean ± SD)	19.8 (4.6)	20.5 (4.7)	23.5 (6.1)	0.04
Preoperative medications	*n* (mean ± SD)	3.1 (0.6)	2.9 (0.4)	3 (0.6)	0.52
Mean deviation	dB(mean ± SD)	−12.6 (7.6)	−8.9 (7.1)	−8.5 (8.3)	0.03
MD < −6	dB(mean ± SD)	10 (22.7)	15 (45.5)	20 (48.8)	
MD −6–−12	dB(mean ± SD)	10 (22.7)	10 (30.3)	8 (19.5)	
MD > −12	dB(mean ± SD)	24 (54.5)	8 (24.2)	13 (31.7)	
VFI	(mean ± SD)	64.1 (25.5)	75.5 (25.5)	76.3 (22.6)	0.01
Cup/disk ratio	(mean ± SD)	0.7 (0.2)	0.7 (0.1)	0.7 (0.1)	0.87
Trabeculectomy	*n* (%)	19 (43.2)	15 (44.1)	21 (51.2)	0.81
Phacotrabeculectomy	*n* (%)	25 (56.8)	19 (55.9)	20 (48.8)	0.81

* BCVA (best corrected visual acuity); POAG (primary open-angle glaucoma); CACG (chronic angle closure glaucoma); SD (standard deviation); dB (decibels).

**Table 2:** Postoperative interventions in all groups.

Postoperative Intervention	MMC (*n* = 44)	(%)	OLO (*n* = 34)	(%)	OLO + MMC (*n* = 41)	(%)	*p*
Massage	3	(8.3)	3	(10.3)	---	---	0.156
Suture release	31	(86.1)	18	(62.1)	17	(45.9)	0.002
Needling	12	(27.3)	2	(6.9)	5	(13.5)	0.016
5-fluorouracil	22	(61.1)	4	(13.8)	6	(16.2)	0.000
Total PI *	74		31		29		
No. patients	38		21		18		

* PI (postoperative interventions).

**Table 3:** Postoperative complications after surgery in all groups.

Complications	MMC (*n* = 44)	(%)	OLO (*n* = 34)	(%)	OLO + MMC (*n* = 41)	(%)	Total %
Choroidal detachment	1	(2.3)	3	(8.8)	2	(4.9)	5
Early wound leak	2	(4.5)	1	(2.9)	8	(19)	9.2
Encapsulated bleb	-	---	1	(2.9)	2	(4.9)	2.5
Hematic Tyndall	-	---	-	---	-	---	---
Hypotony maculopathy	2	(4.5)	3	(8.8)	4	(9.5)	7.6
Hypotony > 1 month	5	(11.4)	6	(17.6)	7	(16.7)	15.2
Transient hypotony	1	(2.3)	-	---	1	(2.3)	1.5
Shallow anterior chamber	2	(4.5)	1	(2.9)	2	(4.9)	4.1

## Data Availability

The original contributions presented in the study are included in the article, further inquiries can be directed to the corresponding author.
